# Women of reproductive age's use of maternal healthcare services and associated factors in Liben district, East Borena zone, Oromia Regional State, Ethiopia

**DOI:** 10.3389/fgwh.2024.1282081

**Published:** 2024-09-03

**Authors:** Mekonnen Desta, Serawit Mengistu, Godana Arero

**Affiliations:** Department of Public Health, Adama General Hospital Medical College, Adama, Oromia, Ethiopia

**Keywords:** Liben district, home delivery, institutional delivery, postnatal care, vaccination, maternal healthcare, service use

## Abstract

**Background:**

One of the most important health interventions for reducing maternal morbidity and death is the use of maternal healthcare services. In Ethiopia, maternal healthcare services are not well utilized, particularly in rural pastoralist communities, despite their significance. Therefore, the purpose of this study was to evaluate the use of maternal healthcare services and the characteristics that are related to it in the East Borena zone. Techniques: In September 2020, a community-based cross-sectional survey was carried out in Liben with 416 randomly selected mothers. Mothers who had given birth within the 12 months before the study comprised the respondents. Questionnaires given by interviewers were used to gather the data. The data were transferred to SPSS version 20 for analysis after being entered into Epi-Info version 4.1 for coding. The Kolmogorov-Smirnov, Hosmer, and Lemeshow goodness of fit tests were employed, along with descriptive statistics. Additionally, multivariate and binary logistic regression analyses were carried out. 95% CI and the odd ratio were used to examine the relationship between the outcome and predictive variables.

**Results:**

At least one prenatal visit was received by 60% of moms. Only 21.2% and 17.5% of women had given birth in a medical facility and made use of early postnatal care services. The use of antenatal care was strongly correlated with maternal education [AOR = 2.43 (95% CI: 1.22–4.89)], decision-making capability [AOR = 2.40 (95% CI: 1.3–23.3)], felt compassionate and respectful treatment [AOR = 0.30 (95% CI: 0.18–0.50)], and intended current pregnancy [AOR = 0.22 (95% CI: 0.12–0.37)]. Moms b/n ages 15–19 had a 3.7-fold higher probability of giving birth in a hospitals than moms b/n ages 35 and 49 [AOR = 1.74 (95% CI: 1.02–3.08)]. Mothers who lived far away were 1.02 times less likely to give birth at a hospital than those who could reach one within an hour (AOR = 1.74;95% CI: 1.02, 3.08). While recent use of antenatal care [AOR = 5.34 (95% CI: 1.96–8.65)], planned current pregnancy, and knowledge of using postnatal care were shown to be strongly correlated with danger indicators [AOR = 2.93 (95% CI: 1.59–5.41)], knowledge of danger signs [AOR = 3.77 (95% CI: 2.16–6.57)] and perceived compassionate and respectful care were significantly associated with institutional delivery.

**Conclusion:**

Overall the prevalence of maternal healthcare services utilization was far below the national and regional targets in the study area. Thus, promoting institutional services, raising community knowledge, empowering women to make decisions, and enhancing the infrastructure of the health sector.

## Introduction

An estimated 2.9 million children die in the first month of life, and 289,000 mothers worldwide die during or shortly after pregnancy and childbirth each year (1). Poor women living in rural regions have a lower likelihood of receiving quality maternity healthcare services in less developed nations (1). Ninety-nine percent of maternal deaths worldwide occur in developing nations. 62% of maternal deaths worldwide occur in Sub-Saharan Africa alone (2). Due to their geographic location, African women are disproportionately at risk of death or disability from pregnancy and childbirth, even though the majority of maternal fatalities are preventable (3).

Maternal healthcare service usage is low, which contributes to the high number of maternal deaths that happen during labor, delivery, and the first few days following childbirth, especially in low- and middle-income countries (4). The health of the mother and the unborn child depends on receiving the right care during pregnancy and delivery facility (4). It is essential to provide professional care, ideally in a medical setting, for expectant mothers, new mothers, and the postpartum period (5). Every woman, without exception, needs expert, trained care when giving birth in a setting that respects her birthing culture, is close to her home, and is appropriate for her level of safety (6). Ethiopia is not an exception to the rule that most low-resource countries have significant challenges related to the low utilization of maternal healthcare services (7). Notwithstanding the government's dedication to provide healthcare facilities to the general public via the Health Extension Service Package (HESP), the nation has encountered difficulties in raising the use of high-quality maternity healthcare services (8). In Ethiopia's remote pastoral areas, healthcare facilities and services are underequipped and of poor quality. The few health centers that are currently in operation lack the necessary supplies, equipment, and basic medications (9). A previous study clearly demonstrated that utilization of available maternal healthcare services is also very low in Brazil (10). According to a recent mini-Ethiopia Demographic Health Survey (EDHS) 2019 report, 70% of rural women received at least one antenatal care (ANC) visit from a skilled provider, including health extension workers. Only 40% and 29% of rural women delivered at health facilities and received early postnatal care, respectively (10). In general, the utilization of maternal healthcare services is a complex behavioral phenomenon influenced by several factors at both individual and community levels. Therefore, to improve maternal and child health, barriers limiting the utilization of maternal healthcare services must be identified and addressed at all levels of the healthcare system. Thus, the purpose of this study was to understand the current status of the utilization of maternal health care services by elucidating the various factors influencing the use of these services in the study area.

## Rationality of study

The use of maternal healthcare services (MHS) is a complicated behavioral phenomenon that involves appropriate postnatal care (PNC) services, the number of prenatal care (ANC) visits that are advised, and the delivery of a child by a skilled birth attendant (SBA) (11). Focused prenatal care lowers healthcare costs in underdeveloped nations by limiting the number of visits for pregnancies that are low-risk. Personalized treatment, early disease detection, quality of visits, and readiness for birth-related issues are prioritized by FANC over the number of clinic visits (12). Many sociodemographic and economic factors, including the woman's age, education, employment status, parity, media exposure, household income, awareness and knowledge of antenatal care services, cultural beliefs, woman's autonomy, availability, and access to health care, are linked to the use of antenatal care (13). Zone, marital status of the woman, husband's educational attainment, occupation, awareness of pregnancy danger signs, interval between births, information source, punctual visits, and transportation issues were all significant factors influencing the use of ANC services for the general population (14, 15).

## Methods

### Study setting, design, and population

Childbearing women who had given birth in the 12 months previous to the survey of residents of Liben district, East Borena Zone, Southern Ethiopia, participated in a community-based, cross-sectional study. Ninety-five (195) of the district's 191,494 inhabitants are female. 19,990 women of reproductive age are thought to reside in the district overall. In the District, there are sixteen health posts and six health centers. The dates of this study's conduct were September 15, 2020–23, 2020.

### Sample size and sampling technique

The required sample size of eligible mothers was determined using a single-population proportion formula. The following assumptions were made: the proportion of institutional delivery in the rural community of the Oromia region, according to the mini-EDHS-2019 report, was 40% (10), with a 95% confidence level, a 5% margin of error, and an expected 10% non-response rate. The final sample size was calculated to be 416. A *p*-value of 40 is incredibly low, suggesting that there is little confidence in the findings and that the null hypothesis is not likely to be rejected. Still, I made an effort to give the sources a lot of thought. In general, it is inappropriate for this investigation at the district level. However, it ought to be controlled throughout the ideation and proposal creation phases.

The participants were selected using the following steps: first, six rural kebeles (the smallest administrative unit) were selected from the 16 kebeles in the district using a simple random sampling technique. The census was carried out in the selected kebeles to identify mothers who had given birth in the year prior to the survey. The proportional distribution of the sample size for each selected kebele was determined. Finally, mothers were selected using a systematic random sampling method.

#### Inclusion and exclusion criteria

Women who had given birth in the last 12 months and had been residents of the Liben district for at least six months were included. I used meticulous study design, including techniques like employing objective measures, to overcome recall bias. Mothers who were critically ill and unable to respond to interviews were excluded.

### Operational definition

#### Antenatal care

Throughout her pregnancy, an expectant mother should receive at least eight ANC visits, according to the recent WHO Focused Antenatal Care (FANC) Framework.

#### Child bearing age

The total of age-specific fertility rates divided by the sum of age-specific rates, weighted by the middle of each age group, where a represents the middle of each age range (17.5, 22.5…49.5) or early 30s and late 20s.

Maternal healthcare services include antenatal, delivery, and postnatal care services. Institutional delivery refers to childbirth either in public or private healthcare facilities attended by skilled attendants such as midwives, nurses, doctors, and health officers Postnatal care refers to care given to mothers after childbirth by healthcare professionals in the first 48 h after delivery at a health facility.

### Data collection tool and method

Quantitative data were collected using a structured and semi-structured questionnaire. The questionnaire was based on a review of relevant previous studies and literature. Face-to-face interviews were conducted at respondents' homes. The main contents of the tool included socio-demographic characteristics, maternal obstetric characteristics, health facility-related factors, and maternal healthcare service utilization.

### Data management and quality

The English questionnaire was revised before being translated into the local tongue and then back into English for uniformity. The tool was pretested on 5% of a comparable population. Six diploma nurses who spoke the local languages well participated in the data collection process. As supervisors, two BSc healthcare experts were hired. During the two days of data collection, all supervisors and data collectors received training based on the principal investigator's guide. The supervisors and primary investigators kept a careful eye on every action and gave the data collectors additional guidance and assistance. Every day, the data were examined for inconsistencies, missing information, incompleteness, and improper answers. Following data cleaning, Epi Data version 4.1 was used to code data, and SPSS version 20 was used for analysis.

### Statistical analysis

Summary statistics of means and percentages were used to describe the study population. Bivariate and multivariate logistic regression analyses were performed to identify associations between independent and outcome variables. Variables with a *p*-value of ≤0.25 on bi-variable regression analysis were further entered into the multivariable binary logistic regression model to control for possible confounding variables. The Hosmer–Lemeshow test was used, and the model adequately fit the data at a *p*-value of >0.05. Multi-collinearity between the independent variables was assessed using a variance inflation factor of <10. Crude and adjusted odds ratios with 95% CI were used to determine the strength of association between the outcome variables and independent variables. Statistical significance was set at *p* ≤ 0.05.

## Results

### Socio-demographic characteristics

A total of 416 women of reproductive age who had given birth in the year before before the survey were interviewed, with a response rate of 100%. Of these respondents, 261 (63%) were aged 20–34 years, with a mean (+SD) age of 26.5(±6.5) years. Of the study participants, 399 (96%) were married. The dominant ethnic group was Oromo, which accounted for 84% of total respondents; 60% were Wake-feta (the indigenous religion of Oromo people) and 32% were Muslim. More than two-thirds (78%) had never attended school and most (85%) were housewives. Regarding decision-making regarding maternal healthcare service utilization, the majority (61%) reported that such decisions were made jointly by husband and wife (Table 1).

**Table 1:** Socio-demographic characteristics of the study participants in Liben district, Southern Ethiopia, September, 2020 (*n* = 416).

Socio-demographic characteristics		Frequency	Percentage
Age of respondents	15–19 years	91	22
	20–34 years	261	63
	35–49 years	64	15
Marriage status	Currently married	399	96
	Divorced & widowed	17	4
Religion	Wake-Feta	250	60
	Muslim	134	32
	Christian	32	8
Ethnicity	Oromo	322	83.7
	Somali	89	21
	Others	5	1.3
Women education	No education	330	78
	Primary & above	86	22
Husband education	No education	270	65
	Primary & above	146	35
Women occupation	House wife	352	84.6
	Merchant	35	8.4
	Civil servant	17	5.1
	Students	12	2.9
Family wealth index	Poor	152	37.6
	Medium	133	32
	Rich	131	31.4
Family size	1–3	29	7
	4–5	128	31
	6 & above	259	62
Decision maker for MHSU	Women alone	60	14
	Husband alone	101	25
	Jointly husband & wife	255	61
Travel time to health facility	<1 h	210	51
	≥1 h	206	49
Access to emergency ambulance	Yes	142	34
	No	274	66

Family wealth index measure using easy-to-collect information on a household's possession of certain goods, such televisions and bicycles, building materials used to construct homes and kinds of water access and sanitary facilities are utilized to create the wealth index.

Justification for journey time to the medical institution: Any mother seeking care shouldn't have to travel more than an hour to a medical facility, per the 5-year health reform policy.

### Obstetric characteristics of respondents

Regarding obstetric history, 167 respondents (40%) became pregnant for the first time before the age of 19. Almost half (51%) had had five or more pregnancies. Fifteen women (3.6%) had experienced a stillbirth. For 244 respondents (59%), their most recent pregnancy was planned and intentional (Table 2).

**Table 2:** Obstetric characteristic of the study participants, Liben district, Southern Ethiopia, September 2020, (*n* = 416).

Variable		Frequency	Percentage (%)
Age at first pregnancy	≥18 years	167	40
	19–24 years	216	52
	≥25 years	33	8
Gravidity	1	56	13
	2–4	149	36
	≥5	211	51
Stillbirth history	Yes	15	3.6
	No	401	96.4
Abortion history	Yes	11	2.6
	No	405	97.4
Planned current pregnancy	Yes	244	59
	No	172	41
Attended ANC in last pregnancy	Yes	249	60
	No	167	40
Frequency of antennal care (*n* = 249)	<4 visits	139	56
	≥4 visits	110	44
First ANC visit (*n* = 249)	Before 16 weeks	61	25
	16–36 weeks	178	69
	After 36 weeks	10	4
Place of last delivery	Home	328	78.8
	Health facility	88	21.2
Utilized postnatal care with 48 h	Yes	73	17.5
	No	343	82.5
Know at least one danger signs	Yes	163	39
	No	253	61
Experienced severe illness in last pregnancy	Yes	105	25
	No	311	75
Felt clients privacy is maintained at HF	Yes	183	44
	No	233	56
Is working time of HF is convenient	Yes	331	80
	No	85	20
Felt compassionate and respectful health care at HF	Yes	219	53
	No	197	47
Main reason for last home delivery (328)	Long distance of HF	156	47
	Wish to deliver with family	58	18
	Prefer TBAs	46	14
	Not trust HF	39	12
	Others	29	9

### Maternal healthcare services utilization

**Figure 1:**
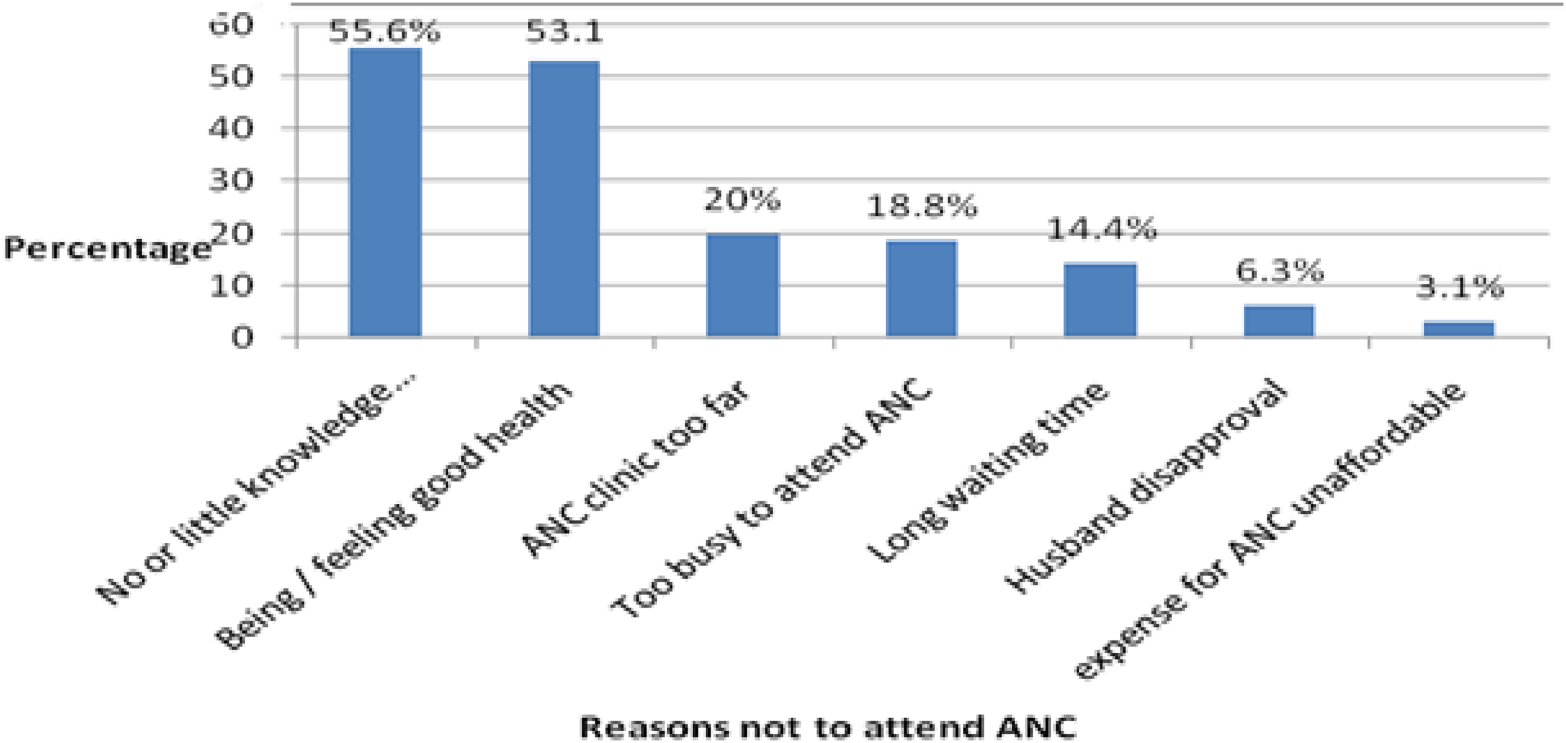
Reasons cited by the study participation for not attending ANC services, Liben district, Southern Ethiopia, September, 2020. ANC, antenatal care; PNC, postnatal care; HF, health facility.

Of all respondents included in the study, 249 (60%) had received at least one ANC service during their last pregnancy. Of these, 138 (55%) had received the service from health extension workers at healthcare clinics. Nearly 25% of the women made their first antenatal visit during the first 16 weeks of pregnancy. Among the ANC service users, 56% had made fewer than four antenatal visits (Table 2). Women cited different reasons for not attending ANC in their recent pregnancies. Little or no knowledge, a feeling of healthiness, a long distance from home to healthcare facilities, work overload at home, and long waiting times for services at healthcare facilities were the major reasons reported for not attending ANC services (Figure 1).

Among respondents, only 88 (21.2%) delivered their baby at a healthcare center/hospital with the assistance of skilled healthcare workers. The majority (78.8%) delivered at home with the assistance of a traditional birth attendant (TBA) and relatives. Women cited different reasons for wanting to deliver at home, such as long distance to healthcare facilities, wishing to deliver in the presence of family, preferring TBAs, and not trusting healthcare facilities (Table 2). Regarding postnatal care service utilization, only 73 (17.5%) of respondents received postnatal care services within 48 h of delivery at a healthcare facility (Table 2).

### Factors associated with maternal healthcare services utilization

According to the multivariate logistic regression analysis, maternal education, decision-making power regarding maternal healthcare service utilization, perceived compassionate and respectful care at healthcare facilities, and planned current pregnancy were significantly associated with the utilization of ANC. Mothers who had completed primary and above education were 3.7 times more likely to utilize ANC services when compared to women with no education (AOR = 3.69; 95% CI: 2.05, 6.62). Mothers who could decide on maternal healthcare services utilization with their husband were 2.4 times more likely to attend ANC visits than women who could not (AOR = 2.4; 95% CI: 1.32, 3.34). Furthermore, women who perceived no compassionate and respectful care at healthcare facilities were 70% less likely to utilize ANC services than women who perceived compassionate and respectful care (AOR = 0.30; 95% CI: 0.18, 0.50). Respondents who had unplanned current pregnancies were 68% less likely to utilize ANC services when compared those who had planned their pregnancies (AOR = 0.78; 95% CI: 0.12, 0.37) (Table 3).

**Table 3:** Bi-variable and multivariable analysis results of maternal healthcare service utilization and its associated factors among childbearing Age of women in liben district, September, 2020.

Factors associated with ANC utilization					
		Antenatal care utilized		COR & 95% CI	AOR & 95% CI
		Yes (*n* = 249)	No (*n* = 167)		
Mother education	No education	179	151	1.00+	1.00+
	Primary & above	70	16	3.69 [2.05, 6.62]*	2.43 [1.21, 4.89]*
Decision maker for MHSU	Women alone	47	13	5.07 [2.44, 5.54]*	4.91 [1.93, 6.56]*
	Both partners	160	95	2.36 [1.47, 3.78]*	2.40 [1.32, 3.34]*
	Husband alone	42	59	1.00+	1.00+
Planned current pregnancy	Yes	184	60	1.00+	1.00+
	No	65	107	0.19 [0.13, 0.30]*	0.22 [0.12, 0.37]*
Compassionate & respectful care	Yes	168	51	1.00+	1.00+
	No	81	116	0.21 [0.14, 0.32]*	0.30 [0.18, 0.50]*
Factors associated with institutional delivery					
** **		Institutional delivery		COR & 95% CI	AOR & 95% CI
		Yes (*n* = 88)	No (*n* = 328)		
Maternal age	15–19 years	35	56	3.01 [1.38, 6.53]*	3.73 [1.53, 6.04]*
	20–34 years	42	219	0.92 [0.44, 1.91]	0.88 [0.39, 1.98]
	35–49 years	11	53	1.00+	1.00+
Travel time to HF	<1 h	55	155	1.86 [1.14, 3.01]*	1.74 [1.02, 3.08]*
	≥1 h	33	173	1.00+	1.00+
Compassionate & Respectful care	Yes	67	152	3.69 [2.16, 6.11]*	2.93 [1.58, 4.40]*
	No	21	176	1.00+	1.00+
Knowledge of danger signs	Yes	60	103	4.68 [2.82, 7.12]*	3.77 [2.16, 6.57]*
	No	28	225	1.00+	1.00+
Factors associated with early PNC utilization					
** **		Postnatal care utilized		COR & 95% CI	AOR & 95% CI
		Yes (*n* = 73)	No (*n* = 343)		
Recent ANC utilization	Yes	68	181	12.1 [3.51, 19.6]*	5.34 [1.96, 8.65]*
	No	5	162	1.00+	1.00+
Planned current pregnancy	Yes	56	188	2.71 [1.51, 4.86]*	2.19 [1.07, 4.41]*
	No	17	155	1.00+	1.00+
knowledge of danger signs	Yes	52	111	5.17 [2.97, 6.01]*	2.93 [1.59, 5.41]*
	No	21	232	1.00+	1.00+

COR, crude odds ratio; AOR, Adjusted odds ratio; CI, confidence interval; MHSU, maternal healthcare service utilization; HF, health facility.

* Significant at *p*-value <0.05, 1.00+ reference category.

The likelihood of delivering at a healthcare institution (health center and hospital) was 3.7 times more likely for mothers aged between 15 and 19 years than for those aged between 35 and 49 years (AOR = 3.73;95% CI: 1.53,6.04). Regarding time to travel to a healthcare facility, mothers who could reach one within an hour were 1.74 times more likely to deliver there (a healthcare center or hospital) compared to those who live further away (AOR = 1.74; 95% CI: 1.02, 3.08). Additionally, mothers who recognized at least one danger sign in pregnancy were 3.7 times more likely to deliver at a healthcare facility (healthcare center or hospital) compared to those who did not (AOR = 3.77; 95% CI: 2.16, 6.57) (Table 2).

Regarding early postnatal care utilization, attending antenatal services in recent pregnancy, planned pregnancy, and knowledge of danger signs in pregnancy were variables that showed an association with postnatal care utilization. Accordingly, those mothers who had attended an ANC visit in their last pregnancy were 5.3 times more likely to utilize early postnatal care services than those who had not (AOR = 5.34; 95% CI: 1.96, 8.65). The likelihood of utilizing early postnatal care services was 2.2 times more likely for mothers who had planned their pregnancy than for those who had not (AOR = 2.19; 95% CI: 1.07, 4.41).

## Discussion

This study set out to assess the level of maternal healthcare service utilization in the Liben district of Southern Ethiopia's Oromia region, as well as the factors that are linked to it. The current study's data indicates that 60% of ANCs were used. This result is consistent with a research that was carried out in the Womberma district (64%) (13). A study carried out in the districts of Enderta (70%) and Kombolcha (86%) revealed increased ANC consumption, which contrasts with the current findings (11, 16). In this study, 21.2% of patients received care through an institutional setting. 78.8% of the women gave birth at home with the help of TBAs and family members. This number is less than that of studies done in the Enderta district (38%), Holeta (61%), and Goba (47%) (11, 17, 18). It is comparable with the study done in rural Kombolicha (20.9%) (16). The survey also showed that only 17.5% of early PNCs were used. This result is consistent with research that was carried out in the rural Jabitena district (20%) (19). It is, however, less than comparable community-based studies carried out in Hossana town (20, 21) and Enderta district (11), where early PNC was obtained by 47% and 51% of women, respectively. The temporal difference between these studies, variations in study settings, and sociodemographic features of the study area could all be contributing factors to this mismatch. Furthermore, the study was carried out in a pastoralist rural district, where the education and decision-making authority of women may be important indicators of the use of healthcare services by mothers. The majority of rural villages have an uneven and small number of healthcare facilities are usually uneven and restricted in rural communities.

According to this study, the use of healthcare services by mothers is correlated with sociodemographic factors including age and educational attainment. Compared to women without any formal education, those who had completed primary school and above had 2.4 times higher odds of using ANC. In addition, moms between the ages of 15 and 19 were 3.7 times more likely than mothers between the ages of 35 and 49 to use institutional delivery. This result is in line with other research from the Enderta, South Omo, and Tigre regions (11, 12, 22).

In addition, moms who could make decisions either by themselves or in conjunction with their husbands were more likely to use ANC than mothers whose husbands made decisions on their own. This outcome is in line with research done in the towns of Holeta and Enderta (11, 17). This could be explained by the fact that women's education plays a critical role in enabling them to be economically independent and to make decisions about maternal healthcare services. This, in turn, enhances their understanding of basic healthcare services and encourages behaviors related to seeking health.

It was observed that pregnant women who used ANC and PNC were more likely to have planned their pregnancies than unplanned ones, probably because of their increased concern for the health and welfare of their unborn child. This result is in line with research findings from Abuna-Gindeberet, Debre-Tabor town, and Wombera (13, 14, 23).

This study also demonstrated that mothers who felt that healthcare providers treated them with compassion and respect had a higher likelihood of using ANC and giving birth in a hospital than mothers who did not. These outcomes are in line with those observed in the districts of Kombolcha and Ambo (16, 21, 24–28). This suggests that women's decisions to utilize or not use a specific type of maternal healthcare service are significantly influenced by the attitudes of healthcare practitioners toward women's healthcare.

According to this study, using a PNC and having understanding of obstetric risk indicators were highly predictive of institutional delivery. Compared to mothers who did not voluntarily indicate any obstetric danger indicators, mothers who were aware of at least one obstetric danger sign were more likely to use institutional delivery and PNC services. This result is consistent with research from the rural Jabitena district (19, 29, 30). This makes sense because early diagnosis, management, and prevention of obstetric danger signals are the main reasons why women and their families should seek medical attention as soon as possible. Awareness of these indicators is a key component in encouraging pregnant women and their families to seek medical attention as soon as possible in order to prevent, identify, and treat obstetric danger indicators.

According to the results of the current study, moms who could get to the hospital in under an hour were 1.74 times more likely to use institutional birth. This result is consistent with research by Enderta and Butajira (11, 31–35). Compared to women seeking ANC, laboring women have less time to get to a hospital. Furthermore, a greater distance between medical institutions and little access to public transit may be linked to higher transportation expenses.

Similar to findings in Hossana, Holeta town, and Ambo district, another noteworthy finding of the current study indicated that recent ANC utilization increased utilization of PNC (17, 20, 24). According to this research, ANC is a crucial starting point for additional maternal healthcare treatments. Pregnant women who use these services are fully informed about the benefits of hospital birth and early postpartum care, as well as the essential follow-up during their pregnancy. But none of the moms who had been for prenatal care made use of PNC (36, 37).

## Strengths and limitations of the study

Because the interviewers conducted the data collection, they were able to clarify any topics that the respondents were unsure of, which increased the accuracy of the information gathered. There was no triangulation of qualitative data collection methods; just the quantitative method was employed. Thus, it is recommended that researchers who are interested in this subject incorporate qualitative methodologies.

## Conclusion

The study area's overall consumption of maternal healthcare services fell well short of the planned expansion and transformation of the health system. The low rate of use of maternity healthcare services in the research region suggests that there is still more to be done to enhance the health of women. The study also showed a strong relationship between ANC utilization and maternal education, decision-making authority, perceived caring and respectful treatment, and current intended pregnancy. While recent antenatal care utilization, planned current pregnancy, and knowledge of danger signs were significantly associated with PNC utilization, mothers' age, time to travel to health facilities, knowledge of danger signs, and perceived compassionate and respectful care were associated with institutional delivery. Consequently, taking into account these established contributing variables and offering education and training on these issues to the community potentially enhance and maintain the use of maternal healthcare services in the community.

## Data Availability

The original contributions presented in the study are included in the article/Supplementary Material, further inquiries can be directed to the corresponding author.
